# Separation of 100 nm-sized nanoparticles using a poly-Lys-modified monolith column†

**DOI:** 10.1039/d4ra07906j

**Published:** 2025-01-30

**Authors:** Masaru Kato, Yui Shirakawa, Yuka Kanai, Shigenori Ota, Nozomi Murayama, Shota Miyazaki, Eiichi Yamamoto, Takashi Takaki

**Affiliations:** a Department of Pharmaceutical Sciences, Division of Bioanalytical Chemistry, Showa University Graduate School of Pharmacy 1-5-8 Hatanodai, Shinagawa-ku Tokyo 142-8555 Japan masaru-kato@umin.ac.jp; b Molecular Analysis Facility, Showa University 1-5-8 Hatanodai, Shinagawa-ku Tokyo 142-8555 Japan; c Department of Pharmaceutical Sciences, Division of Bioanalytical Chemistry, School of Pharmacy, Showa University Japan; d GL Sciences Inc. 237-2 Sayamagahara, Iruma Saitama 358-0032 Japan; e Division of Medical Devices, National Institute of Health Sciences 3-25-26 Tonomachi, Kawasaki-ku Kawasaki City Kanagawa 210-9501 Japan; f Center of Electron Microscopy, Showa University Japan

## Abstract

Nanoparticles (approximately 100 nm in diameter) composed of lipid layers containing drugs or biologically active substances are attracting increasing attention in various fields, including medicine, as well as for signal transduction between cells. However, the separation of such nanoparticles *via* conventional HPLC is challenging, often resulting in the clogging and collapse of nanoparticles, as well as a low separation efficiency. Thus far, no HPLC column capable of efficiently separating two types of 100 nm-sized nanoparticles in a short time has been reported. In this study, a poly-Lys-modified monolithic column was prepared for nanoparticle analysis *via* HPLC using anticancer drug-encapsulated nanoparticles (Doxil®) and small extracellular vesicles (sEVs) to examine their elution behaviors. The zeta potentials of Doxil® and the sEVs were −24.4 and −45.5 V, respectively. A column with a low surface coverage (0.96 mg mL^−1^) of poly-Lys adsorbed the nanoparticles but did not elute them, whereas a column with a high surface coverage (2.06 mg mL^−1^) of poly-Lys retained these nanoparticles owing to the ion-exchange effect; sEVs with highly negative charges were strongly retained in the column. Using gradient elution with different 2-amino-2-hydroxymethyl-1,3-propanediol concentrations in the mobile phase, the two types of nanoparticles (Doxil® and sEVs) were eluted and successfully separated within 10 min. Thus, the developed column is a valuable tool for evaluating the safety and performance of larger-sized nanoparticles.

## Abstract 2

Development of an effective HPLC column for rapid and efficient separation of 100 nm nanoparticles, such as nanomedicines and small extracellular vesicles, which are of interest in various fields.

## Graphical abstract

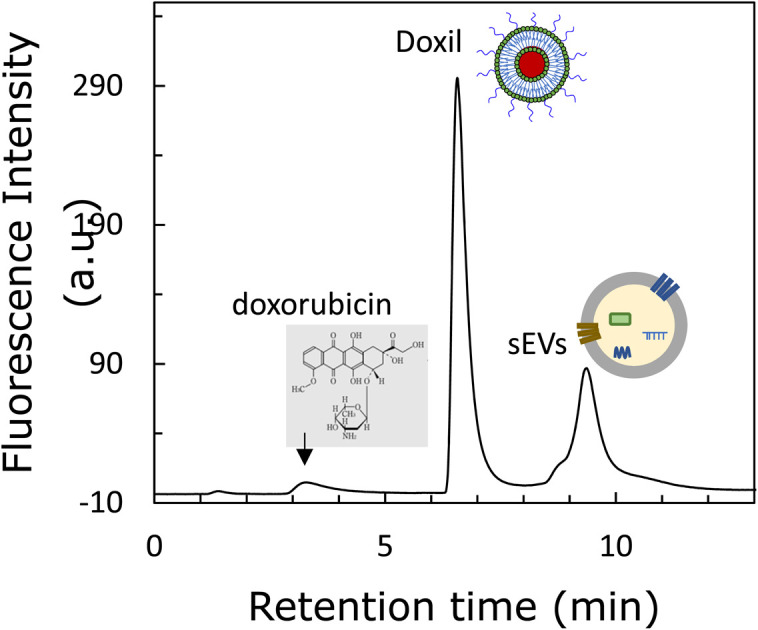

## 1. Introduction

Nanoparticles have a size of one to several hundred nanometers and are currently used in various fields.^1–5^ Because the properties of nanoparticles are greatly influenced by their size, controlling the size of nanoparticles is important in their use. The smaller the nanoparticle, the larger the surface area ratio, and the more pronounced are the nano characteristics. A larger size allows for more substances to be encapsulated in the nanoparticle. In addition, considering the pharmacokinetics of nanoparticles, if they are too small, they will be excreted by the kidneys, and if they are too large, they will be eliminated by the immune system. Nanoparticles sized between 10 and 200 nm circulate in the body for long durations *via* the bloodstream.^6^ The properties of nanoparticles change significantly depending not only on their size, but also on their shape and surface condition.^7–9^ Therefore, to use nanoparticles safely and effectively, it is important to have a method for separating and purifying nanoparticles based on their physical properties.

HPLC is widely used for separating and purifying substances, and its application to nanoparticles is progressing.^10,11^ Because the separation efficiency in chromatography depends largely on the diffusion rate of the analyte, nanoparticles with low diffusion rates tend to have broad peaks, making complete separation difficult. However, nanoparticles as small as a few nanometers can be effectively separated using a standard reversed-phase HPLC column.^12,13^ For such nanoparticles, good separation can be achieved by using the mesopores (pore diameter of approximately 10 nm) of the packed particles in the HPLC column. However, nanoparticles sized ∼10 nm or more cannot fit into the mesopores of the packed particles of typical HPLC columns. Therefore, separation is performed using a size-exclusion chromatography (SEC) column with large pores or a column packed with non-porous particles.^14,15^ When the particle size becomes even larger—100 nm or more—the nanoparticles cannot pass through the interstitial pores between the packed particles, and the possibility of the nanoparticles clogging the column increases, making it difficult to use existing HPLC columns.

Consequently, the development of columns that can pass and separate nanoparticles sized approximately 100 nm or more without clogging is underway. Such columns must possess pores large enough for the nanoparticles to pass through. There are two main methods to create large pores. One is to increase the particle size, which increases the size of the interstitial pores between the packed particles, and the other is to use a monolith column.^16–20^ In a particle-packed column, the particles are packed under high pressure, approaching a close packing structure. Therefore, the size of the interstitial pores between the particles is proportional to the particle size. However, the larger the particle size of the packed particles, the lower the separation efficiency, making it difficult to achieve good separation. In fact, when nanoparticles were separated by SEC in a column packed with large particles (13 μm in size), nanoparticles of several hundred nanometers were eluted in order of their size; however, complete separation of each peak was not possible.^19^ In a monolith column, the size of the stationary phase and the pore size of the through-pores can be adjusted independently, making it possible to create a monolith structure with through-pores of several micrometers that can pass large nanoparticles. Monolith columns were previously modified with a lectin that recognizes the glycan of exosomes, resulting in the successful selective separation and purification of exosomes of approximately 100 nm in size from a mixed sample.^20^ However, there have been no reports of the complete separation of two or more types of nanoparticles of approximately 100 nm in size in a short period of time based on their physical properties.

In this study, we prepared two types of poly-Lys modified monolith columns with different coverage amounts and investigated the elution profiles of two similarly sized (100 nm) nanoparticles (anticancer drug-encapsulated nanoparticles (Doxil®) and small extracellular vesicles (sEVs)) to develop a nanoparticle separation column based on the surface charge of the nanoparticles. The nanoparticles were well separated in approximately 10 min through a gradient elution of the Tris concentration using a column with a high coverage of poly-Lys.

## 2. Experimental

### 2.1 Chemicals

Cationic silica particles (InertSep®SAX: particle size, 45 μm; pore size, 6 nm; carbon content, 7%; and ion-exchange capacity, 0.7 meq g^−1^) were obtained from GL Sciences (Tokyo, Japan). Sodium chloride, 10× d-phosphate buffer saline (DPBS)(−), 2-amino-2-hydroxymethyl-1,3-propanediol (Tris), adenosine 5′-mono phosphate, cytidine 5′-monophosphate, 3,3′,5,5′-tetra methylbenzidine solution (TMB; for Microwell), albumin from bovine serum (BSA), and Cohn fraction V (pH 7.0) were obtained from FUJIFILM Wako Pure Chemical Corporation (Osaka, Japan). Fetal bovine serum (FBS) was obtained from DS Pharma Biomedical Co., Ltd (Osaka, Japan). Doxil® (doxorubicin HCl liposome) was purchased from Janssen Pharmaceutical K.K (Tokyo, Japan) and used without further purification. Doxorubicin was purchased from Tokyo Chemical Industry Co., Ltd (Tokyo, Japan). Tetraethoxysilane (TEOS) was obtained from Shin-Etsu Chemical (Tokyo, Japan). The red fluorescent cell linker (PKH26), polyethylene oxide, and poly-Lys were obtained from Sigma-Aldrich, Inc. (St. Louis, MO, USA). Water was purified with a Milli-Q apparatus (Millipore, Bedford, MA, USA).

An anti-mouse IgG antibody and horseradish peroxidase (HRP) were obtained from Vector Laboratories, Inc. (Burlingame, CA, USA). Anti-CD9, anti-CD63, anti-CD81, and anti-apolipoprotein B were obtained from Cosmo Bio Co., Ltd (Tokyo, Japan).

### 2.2 Preparation of poly-Lys-modified column

The column was prepared by forming a monolith silica rod,^21^ molding it into a column,^21^ and modifying it with poly-Lys^22^ based on a previously reported method. Briefly, TEOS was added to a 1 M aqueous solution of nitric acid in the presence of polyethylene oxide with an average molecular weight of 100 000 and d-sorbitol. The mixture was then stirred for 15 min at 25 °C. The solution was maintained at 40 °C for gelation and aged for 15 h. The aged gel was immersed in a 1.5 M aqueous urea solution at 110 °C for 20 h. After drying at 40 °C for 24 h, the gel was heated at 600 °C for 5 h. A 50 mm-thick silica rod with a diameter of 3.0 mm was cut. The poly-Lys-modified column was prepared by immersing the silica monolith in a poly-Lys solution using a previously reported method.^22,23^ The through-pore and mesopore sizes of the monolith were 1 μm and 50 nm, respectively, and its surface area and porosity were 40 m^2^ g^−1^ and 80%, respectively.

### 2.3 Evaluation of monolithic column

The modified amount of poly-Lys on the monolith column was calculated by measuring the amount of poly-Lys reduced in the modification solution using a UV spectrometer (U-2910, Hitachi, Tokyo, Japan). AutoPore V (Micrometrics, Norcross, GA, U.S.A.) was used to determine the sizes of the through- and mesopores and surface area of the monolith column.

### 2.4 sEV sample preparation

sEVs were purified from FBS using a previously reported method.^24^ Briefly, 100 mg of cationic particles and 300 μL of FBS were added to an Eppendorf tube and mixed using a vortex mixer. The particles were washed four times with 300 μL of PBS, and the resulting supernatant was discarded. The sEVs were eluted with a 200 mM aqueous NaCl solution (100 μL). The obtained sEV solution was mixed with the PKH26 solution and stored for 2 h at room temperature (approximately 20 °C). The sample solution was filtered using Millex®-LH (0.45 μm, Merck) prior to HPLC.

### 2.5 sEV validation

The purified sEVs were validated using three positive markers (CD9, CD63, and CD81) and one negative protein marker (apolipoprotein B) according to established guidelines from the International Society for Extracellular Vesicles (ISEV).^25^ Protein detection was performed using the ELISA method described in our previous paper.^24^ First, 100 mg cationic particles and 300 μL FBS were added to an Eppendorf tube and mixed using a vortex mixer. The solution was incubated for 30 min at room temperature (approximately 20 °C) after the addition 100 μL of 1 mM BSA, and then washed five times using 300 μL of 25 mM phosphate buffer (pH 7). Next, 100 μL of an antibody solution was added, and the mixture was incubated for another 30 min. The obtained mixture was washed five times with the phosphate buffer. The solution was incubated for an additional 30 min after the addition of 100 μL of an HRP solution and 300 μL phosphate buffer. The solution was then washed five times with the phosphate buffer, after which 200 μL TMB and 100 μL phosphate buffer were added to the solution and the mixture was incubated for 30 min. Finally, 100 μL of 500 mM sulfuric acid was added to the sample solution to terminate the reaction. The sample solution (200 μL) was dispensed into a 96-well plate and thoroughly mixed on a plate shaker for 30 min. The absorbance at 450 nm was measured using a multiplate reader. The results are shown in ESI Fig. 1.†

### 2.6 HPLC

HPLC (Hitachi, Tokyo, Japan) was performed with a Chromaster 5110 pump, a 5210 autosampler, an FL detector L-7480, and an HPLC system organizer. A monolith column (50 mm × 3.0 mm I.D.) was used at a flow rate of 0.5 mL min^−1^. The injection volume was 10 μL and the detection wavelengths were set at 550 nm for excitation and 580 nm for emission.

### 2.7 Nanoparticle size analysis *via* dynamic light scattering

The Nanotrac Wave dynamic light scattering (DLS) instrument (Microtrac BEL Corp., Osaka, Japan) employed in this study is described elsewhere.^26^ The measurements were carried out at room temperature (approximately 20 °C) using a 780 nm laser beam. The sample solution was diluted 40-fold before the measurement. A minimum of three replicate measurements was performed for each sample, and sample sizes of 20 μL were employed throughout.

### 2.8 Zeta potential measurement *via* nanoparticle tracking analysis

The zeta potential of the nanoparticles was measured *via* nanoparticle tracking analysis (NTA) using a ZetaView Particle Tracking Analyzer (MicrotracBEL Corp. Osaka, Japan). All samples were diluted with a 50 mM NaCl solution. NTA data were recorded and analyzed at 11 positions while maintaining the temperature at 25 °C. A minimum of eight replicates were acquired for each sample.

### 2.9 Morphology observation of nanoparticles using transmission electron microscopy

Transmission electron microscopy (TEM) samples were prepared by dropping approximately 10 μL of the sample dispersion onto a Formvar carbon film on copper grids (300 mesh; Nisshin EM, Tokyo, Japan). The grids were negatively stained with 2% (wt/vol) uranyl acetate. Images were obtained using an H-7600 transmission electron microscope (Hitachi Co. Ltd, Tokyo, Japan).

## 3. Results and discussion

### 3.1 Properties of the nanoparticle sample

**Fig. 1:**
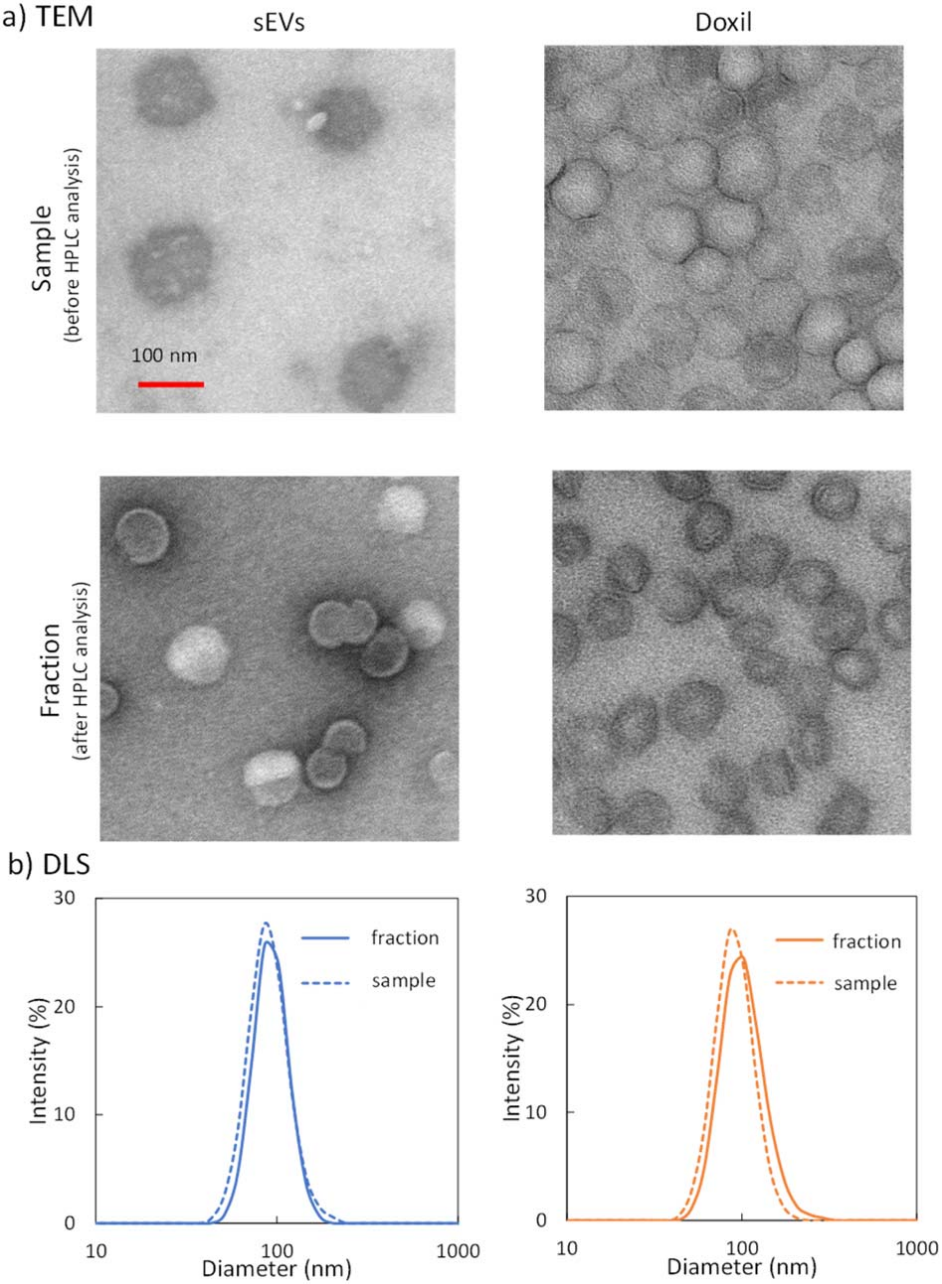
(a) Transmission electron microscopy images of the small extracellular vesicles (sEVs) (left) and Doxil® (right). Images on the top row and middle row were obtained before and after HPLC, respectively. (b) Dynamic light-scattering analysis of sEVs and Doxil® before (dotted line) and after (solid line) HPLC.

The physical properties of the nanoparticles used in this study were investigated first. Fig. 1a shows the TEM images of the sEVs (top left) and Doxil® (top right). Fig. 1b shows the particle size distribution of the solutions of sEVs (left) and Doxil® (right) measured *via* DLS as a dotted line. As shown in Fig. 1, both particles are spherical and of similar sizes. The particle sizes of sEVs and Doxil® were 81.4 ± 2.0 and 82.1 ± 1.4 nm, respectively (Table 1). NTA revealed that the zeta potentials of the sEVs and Doxil® were −45.5 ± 4.8 and −24.4 ± 2.1 V, respectively (Table 1), with the sEVs exhibiting a higher negative charge. Although the two particles were nearly identical in size, their zeta potential values differed significantly. The larger variability of the zeta potential values (Table 1) for sEVs may be attributable to the heterogeneity of their origins.

**Table 1:** Size, polydispersity index (PDI), and zeta potential of the nanoparticles

	Size (nm)	PDI	Zeta potential (V)
sEV	81.4 ± 2.0	0.184	−45.5 ± 4.8
Doxil®	82.1 ± 1.4	0.132	−24.4 ± 2.1

### 3.2 Effect of coverage amount of poly-Lys

**Fig. 2:**
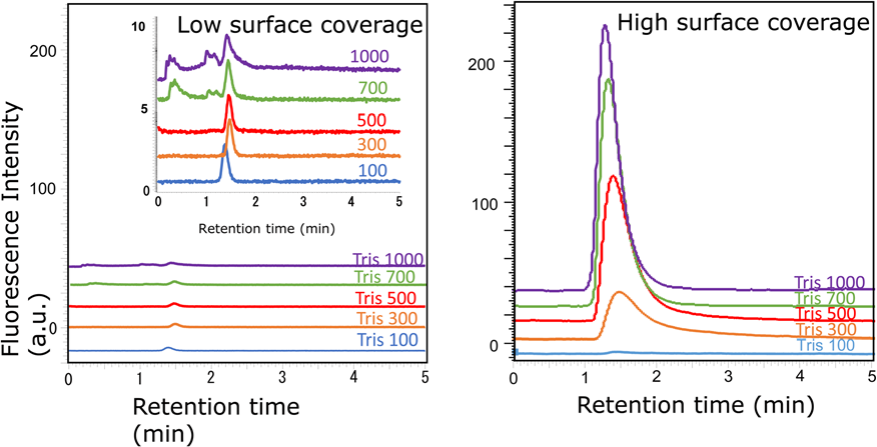
Chromatograms of sEVs obtained using two types of poly-Lys modified columns with a Tris buffer (pH 8) at different concentrations (mM).

In our previous study on nanomedicines, the elution of Doxil® in a monolithic column with low surface coverage was difficult unless an organic solvent was added to the mobile phase.^26^ Doxil® was adsorbed to the remaining silanol groups of the stationary phase. Therefore, two poly-Lys-modified monolithic columns with different surface coverage amounts were prepared and their elution behaviors were examined. The surface coverage amounts per mL of the monolith column in the low- and high-coverage columns were 0.96 and 2.06 mg mL^−1^, respectively. Fig. 2 shows the chromatograms when Tris buffer (pH 8) was used as the mobile phase; its concentration was varied in the range of 100–1000 mM. A magnified image is also shown for the low-coverage column, where no large peaks are detected. No large peaks were detected by the low-coverage column, regardless of the Tris concentration. This is consistent with the results observed with Doxil® in our previous studies.^26^ Furthermore, Doxil® did not elute on this column. In the high-coverage column, although no peak was detected when the Tris concentration was 100 mM, a large peak was eluted around 1.5 min when the Tris concentration was more than 300 mM. This significant change in the sEV elution behavior was attributed to the coverage amount of poly-Lys and the effect of residual silanol groups on the stationary phase. As the surfaces of both sEVs and Doxil® are composed of a lipid bilayer membrane, the surface membrane is assumed to be strongly adsorbed to the residual silanol of the stationary phase. Because the elution behavior of the sEVs changed depending on the Tris concentration in the high-coverage column, the sEVs were retained by the ion-exchange effect of poly-Lys on the monolith, and their elution could be controlled by adjusting the Tris concentration of the mobile phase. Accordingly, a high-coverage column was chosen for further separating the nanoparticles.

### 3.3 Elution profile of sEVs

**Fig. 3:**
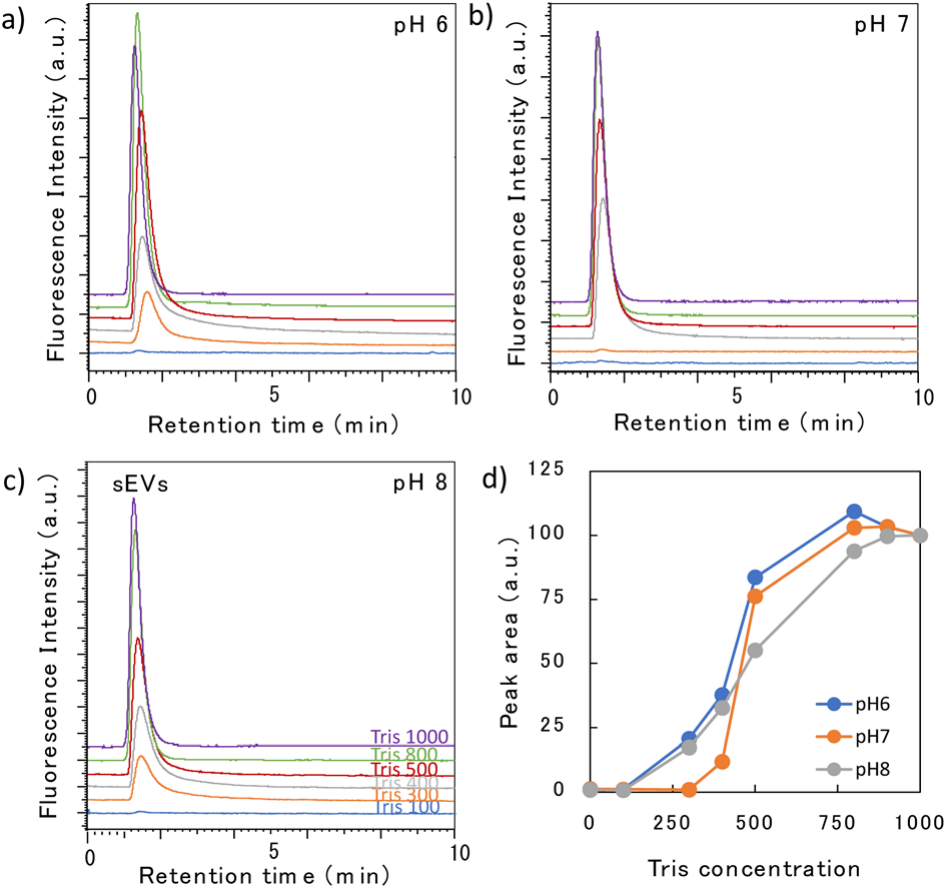
(a–c) Chromatograms of sEVs obtained using a high-surface-coverage poly-Lys column at different pH levels and Tris concentrations (mM). (d) Relationship between the Tris concentration and peak area at different pH levels.

Subsequently, we examined the elution behavior of sEVs when the pH and Tris concentration were varied (Fig. 3a–c). At a mobile phase pH between 6 and 8, no elution occurred with 100 mM Tris buffer; however, when the Tris concentration was 300–400 mM or higher, elution occurred after 1.5 min. Fig. 3d shows the change in the peak area with changing Tris concentration. The peak area when 1000 mM Tris was used in the mobile phase was set as 100%, and the area at each concentration was plotted as a relative value. The peak intensity gradually increased as the Tris concentration increased, but remained constant in the range of 700–800 mM. This suggests that at low concentrations, only some sEVs were eluted, and the proportion of sEVs eluted increased as the Tris concentration rose. The gradual increase in the peak intensity of sEVs with increasing Tris concentration is likely due to the nonuniform surface charge of intracellularly produced sEVs, which varied among vesicles; this variation indicated that the Tris concentration required for elution differs among the vesicles. No significant change in the elution behavior was observed with changes in the pH. This suggests that in this pH range, there is no significant change in the electrostatic interaction between the phosphate groups of sEVs and the amino groups of poly-Lys of the stationary phase.

### 3.4 Elution profile of Doxil®

**Fig. 4:**
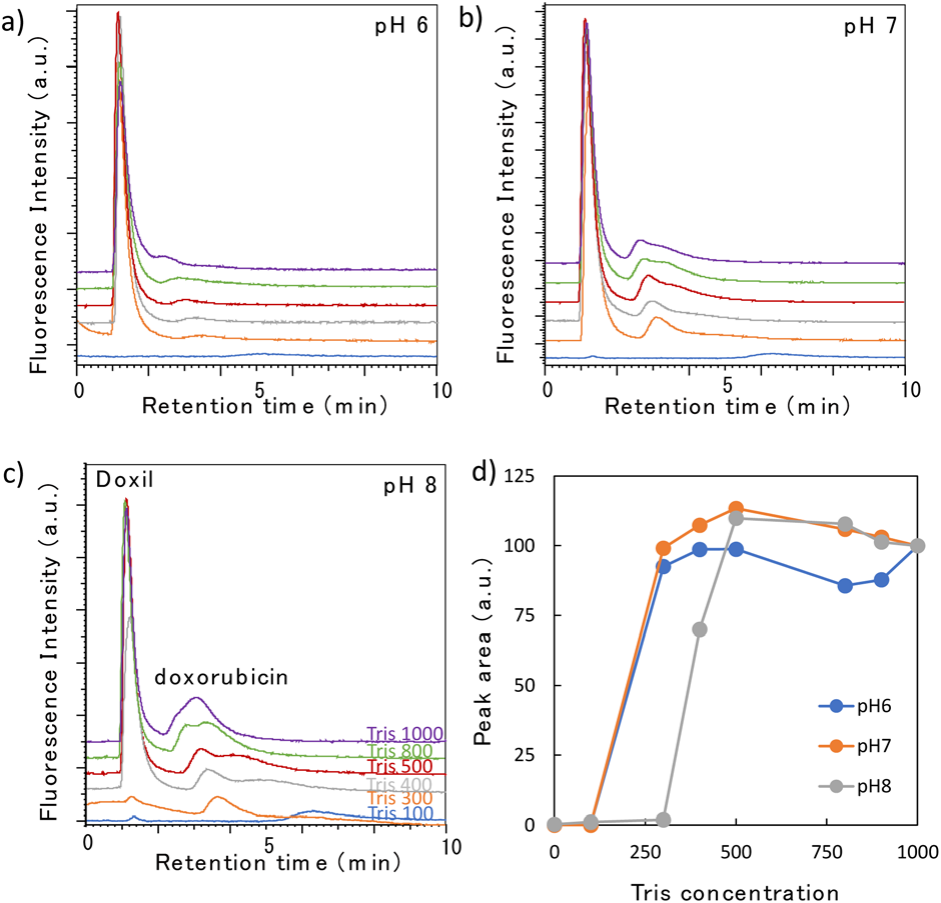
(a–c) Chromatograms of Doxil® obtained using a high-surface-coverage poly-Lys column at different pH levels and Tris concentrations (mM). (d) Relationship between the Tris concentration and peak area at different pH levels.

Doxil®, the first FDA-approved nanomedicine, comprises a lipid bilayer similar to that of sEVs, but its composition is different, and its surface is modified with polyethylene glycol chains. Hence, we compared the elution behaviors of Doxil® and sEVs under the same conditions. Doxil® contains liposome-encapsulated doxorubicin as an anticancer drug. When the Doxil® sample solution was analyzed, in addition to the Doxil® peak at 1.5 min, a small peak of the free doxorubicin that leaked from Doxil® was detected at 3–4 min (Fig. 4a–c). When the amount of doxorubicin contained in Doxil® was analyzed, a peak approximately 1500 times stronger than the Doxil® peak was detected (ESI Fig. 2†). As the doxorubicin peak detected here is very small, we inferred that its leakage was not significant. The large change in the peak intensity for doxorubicin was due to quenching during encapsulation, leading to a loss in fluorescence. By contrast, when doxorubicin was released from the nanoparticles, the quenching effect was lifted, resulting in strong fluorescence. Although the elution behavior of Doxil® remained largely unaffected by the pH, it was significantly affected by the Tris concentration. The Tris concentrations at which the Doxil® peak appeared were 300 mM at pH 6 and 7, and 400 mM at pH 8. This dependence was attributed to the Tris buffer being prepared by mixing 2-amino-2-hydroxymethyl-1,3-propanediol and hydrochloric acid; even if the concentration of the former was the same, the chloride ion concentration in the buffer increased as the pH decreased. Therefore, the lower the pH, the stronger the elution force owing to the ionic concentration of the mobile phase. A similar phenomenon was observed in our previous studies.^22^ The peak intensity increased rapidly once the peak was detected and then remained almost constant (Fig. 4d). For example, at pH 7, a peak was detected at 300 mM, and even when the concentration increased, the peak intensity hardly changed. Most samples of Doxil® required approximately the same Tris concentration for elution because Doxil® is prepared using precise amounts of recognized compounds following strict manufacturing procedures, resulting in a highly homogeneous formulation.

When the repeatability of the column was examined, the relative standard deviations (RSDs) of the elution times of Doxil® and free doxorubicin (*n* = 5) were 1.00% and 1.30%, respectively. The RSDs of their peak areas were 1.88% and 3.53%, respectively. The poly-Lys-covered columns can be used for over a year and a half, indicating their enhanced durability compared to triethylammonium-modified columns.^27^ Therefore, the developed column is considered practical.

### 3.5 Size and morphology analysis of the eluted nanoparticles

Although nanoparticles were eluted by changing the mobile phase conditions, this may be attributable to fragments generated by the collapse or decomposition of nanoparticles in the column. Therefore, peak fractions were collected, and the components were evaluated using electron microscopy and DLS. Fig. 1a shows the TEM images of sEVs (left) and Doxil® (right). Images on the top were obtained before HPLC, and those on the middle were obtained after HPLC. No major morphological changes in the shape of either particle were observed before and after the analysis. Subsequently, the size distribution of the nanoparticles before and after passing through the column was measured using DLS (Fig. 1b). The size distribution peaks of both nanoparticles before (dotted line) and after (solid line) HPLC analysis almost overlapped. The average particle sizes of the sEVs before and after HPLC were 81.4 ± 2.0 and 84.5 ± 0.3 nm, respectively. Similarly, the average particle sizes of Doxil® were 82.1 ± 1.4 and 90.6 ± 2.5 nm, respectively. The slight increase in the average particle size of both particles can be attributed to the removal of small particles by HPLC or the reduction in electrostatic repulsion between particles in the mobile phase. However, the peaks of both nanoparticles overlapped, suggesting that the morphology of most particles did not change during the analysis.

### 3.6 Separation of sEVs and Doxil® using gradient elution

**Fig. 5:**
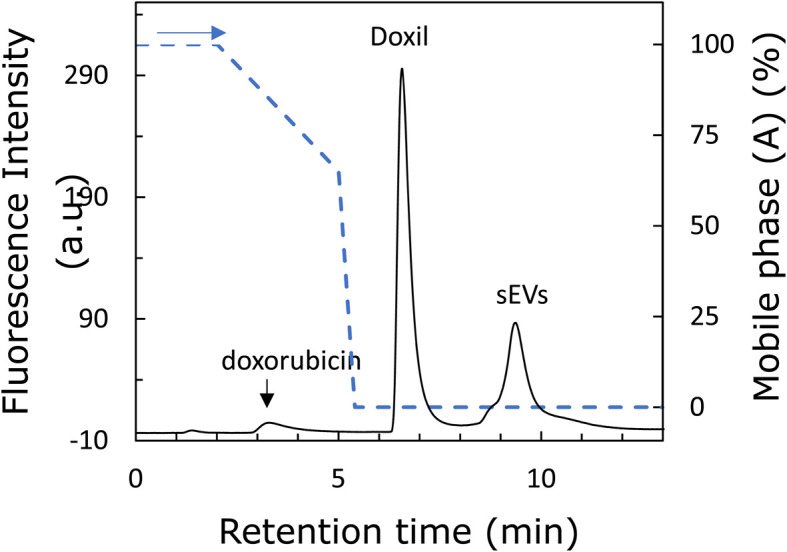
Chromatogram of a mixed solution containing both sEVs and Doxil®. Left *y*-axis: fluorescence intensity. Right *y*-axis: mobile phase (A) ratio, mobile phase (A): water; (B): 1 M Tris buffer (pH 8); gradient program: (A) 100% (0 → 2 min), 100 → 65% (2 → 5 min), 65 → 0% (5 → 5.4 min), 0% (5.4 → 11 min).

The two types of nanoparticles passed through the column intact and were found to have different affinities for the stationary phase; therefore, an attempt was made to separate them. Fig. 5 shows the chromatogram of the separation of Doxil® and sEVs using a mobile phase with pH 8. The peaks of free doxorubicin, Doxil®, and sEVs were eluted at approximately 3.5, 6.5, and 9.5 min, respectively. The elution time of doxorubicin was not significantly affected by the Tris concentration. Therefore, when the Tris concentration was low, the two nanoparticles were retained, while doxorubicin eluted first. In addition, a small shoulder was detected in front of the sEV peak. This is likely because of the diverse particle sizes and surface modification groups among the sEVs, with the weakly retained sEVs eluting first. Thus, the two nanoparticles were well separated. The sizes of the two nanoparticles were almost the same; therefore, we expected the zeta potential to be the cause of separation of the two nanoparticles. sEVs are more negatively charged, implying that they have a stronger affinity for the amino groups of the stationary phase. A comparison of Fig. 3c and 4c reveals that the Tris concentration required for desorption from the stationary phase is higher for sEVs, suggesting that sEVs are strongly retained on the stationary phase by the anion-exchange effect. This suggests that anion exchange is involved in the separation of the two nanoparticles. The fact that anion-exchange is working in this column can also be confirmed by the fact that the elution behavior of nucleic acids (ESI Fig. 3†) is similar to the reported elution behavior of nucleic acids by ion-exchange columns.^28,29^ SEC and nanoparticle exclusion chromatography^30–32^ are both HPLC-based methods for nanoparticle separation. However, these methods cannot separate nanoparticles based on their surface states.^16–19,33^ In addition, analyzing 100 nm-sized nanoparticles *via* SEC is time-consuming due to the lower diffusion rates of such nanoparticles compared to small molecules. In contrast, the poly-Lys-modified column successfully recognized two different nanoparticles by characteristics such as the surface charge in approximately 10 min, demonstrating its potential application in nanoparticle analysis. Therefore, this technology is expected to be useful in applications such as the separation of sEVs and nanomedicines in blood samples, which are crucial in the medical field.^34^

## 4. Conclusions

In this study, we prepared a poly-Lys-modified monolithic column and used it for the separation of nanomedicines and sEVs based on their surface state using HPLC. Our results showed that nanoparticles were retained *via* the ion-exchange effect and eluted with increasing Tris concentrations in the mobile phase. In addition, the nanoparticles hardly collapsed or aggregated during column separation. Using gradient elution, the two nanoparticles were successfully separated within 10 min. Surface charges were involved in the separation of nanoparticles, and nanoparticles with higher negative zeta potentials were retained in the stationary phase and required higher Tris concentrations for elution. To the best of our knowledge, no HPLC methods for separating nanoparticles of approximately 100 nm in a short time existed previously; therefore, our method is a valuable tool for the analysis and evaluation of nanoparticles used in various fields. In recent years, the medical field has increasingly used nanomedicines, which contain approximately 100 nm-sized particles designed to encapsulate drugs. However, accurate kinetic analysis is important for their safe and effective use. In addition, it is essential to separate and measure sEVs present in the body from administered nanomedicines to accurately measure the blood concentration of nanomedicines. Furthermore, the presence of a small shoulder preceding the sEV peak suggests that the poly-Lys column may be capable of separating sEVs with distinct zeta potentials. Therefore, developing a simple and rapid measurement method for this purpose is extremely important.

## Data availability

The authors declare that the data supporting the findings of this study are available within the paper and its ESI files.† Should any raw data files be needed in another format they are available from the corresponding author upon reasonable request.

## Author contributions

Masaru Kato: conceptualization, data curation, funding acquisition, investigation, project administration, writing – original draft, and writing – review & editing. Yui Shirakawa: data curation, formal analysis, and writing – original draft. Yuka Kanai: data curation, formal analysis, and writing – original draft. Shigenori Ota: resources, and writing – original draft. Nozomi Murayama: resources, and writing – original draft. Shota Miyazaki: resources, and writing – original draft. Eiichi Yamamoto: funding acquisition, investigation, writing – original draft, and writing – review & editing. Takashi Takaki: data curation, formal analysis, and writing – original draft.

## Conflicts of interest

There are no conflicts to declare.

## Supplementary Material

RA-015-D4RA07906J-s001
