# Supplementary material for: Separation of 100 nm-sized nanoparticles using a poly-Lys-modified monolith column
Source: RSC Adv. 2025 Jan 30;15(5):3147–53. doi: 10.1039/d4ra07906j (PMC11781079; doi:10.1039/d4ra07906j)
Supplement: RA-015-D4RA07906J-s001 [file RA-015-D4RA07906J-s001.pdf]

Supplementary Information (SI) for RSC Advances.
This journal is © The Royal Society of Chemistry 2025

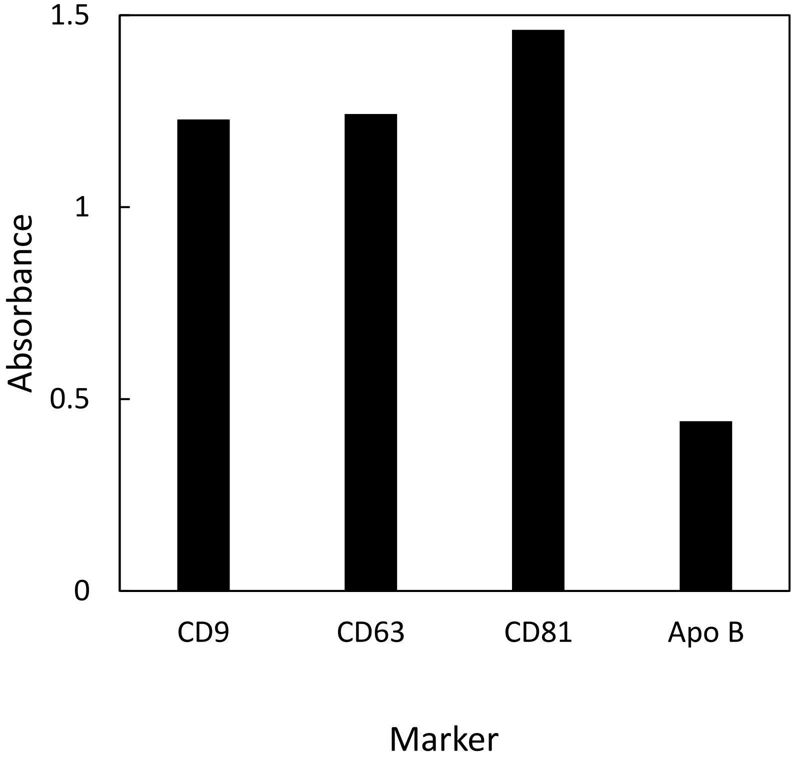

Fig. S1 sEV validation by ELISA

Supplementary Figure 1

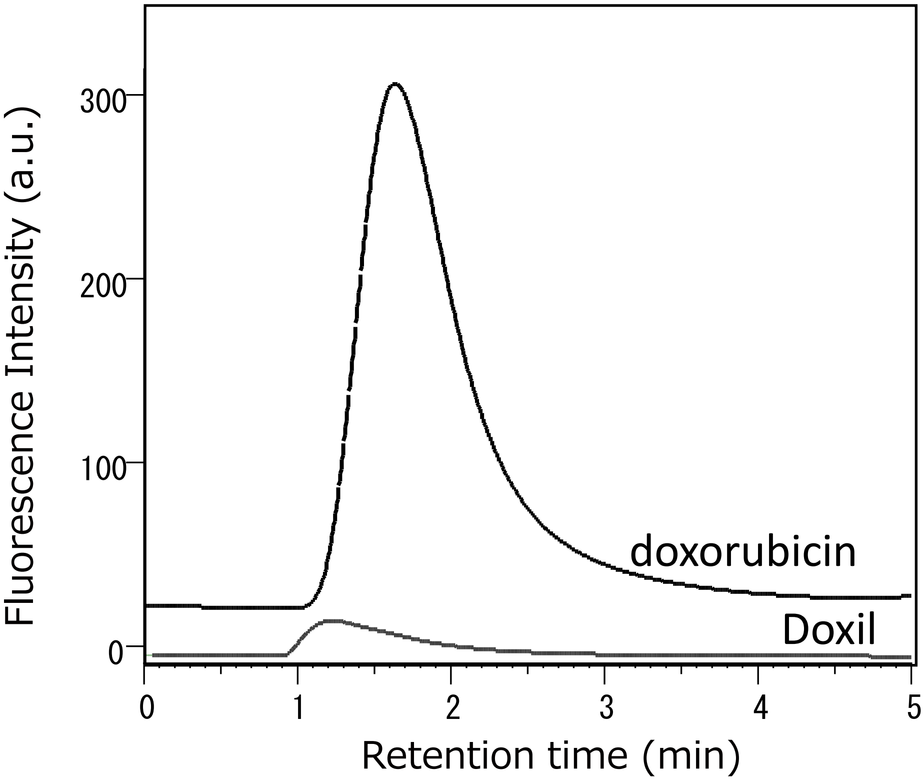

Fig. S2 Chromatograms of doxorubicin and Doxil at the same dose of doxorubicin

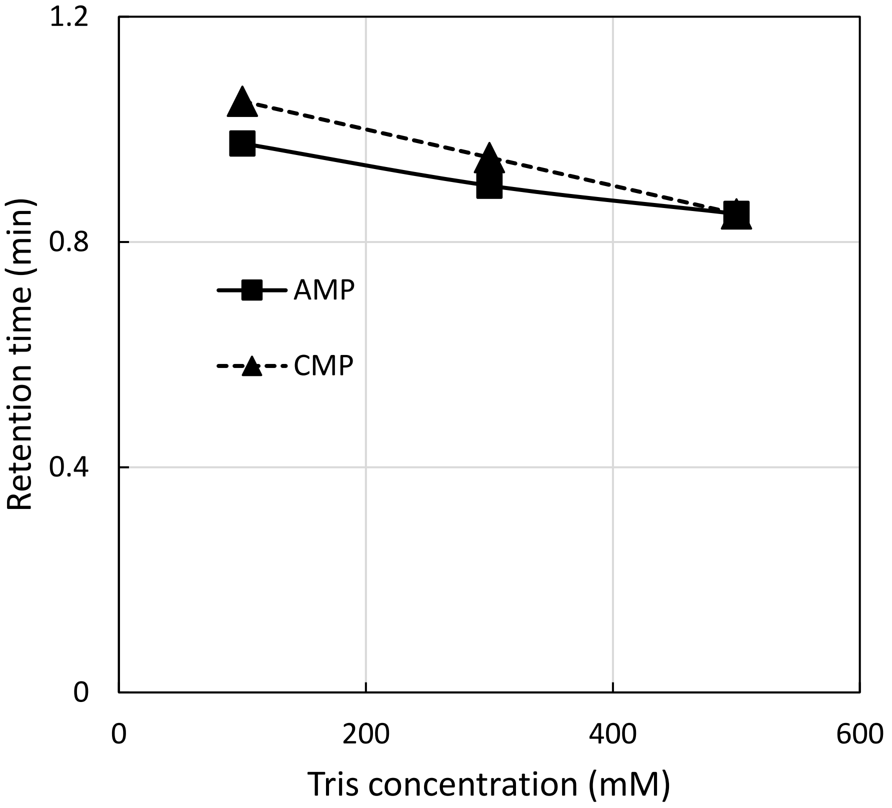

Fig. S3 Effect of Tris concentration on the retention times of adenosine 5′-monophosphate (AMP) and cytidine 5'-monophosphate (CMP).
Mobile phase: Tris buffer (pH7)
